# Editorial: Promoting Environmental Health, Sleep, and Nutrition Through Chronobiological Approaches

**DOI:** 10.3389/fpubh.2021.780705

**Published:** 2021-12-10

**Authors:** Elaine C. Marqueze, Cibele A. Crispim, Claudia R. C. Moreno

**Affiliations:** ^1^Public Health Graduate Program, Catholic University of Santos, Santos, Brazil; ^2^School of Medicine, Federal University of Uberlândia, Uberlândia, Brazil; ^3^Department of Health, Life Cycles and Society, School of Public Health, University of São Paulo, São Paulo, Brazil; ^4^Psychology Department, Stress Research Institute, Stockholm University, Stockholm, Sweden

**Keywords:** chronobiology, chrononutrition, sleep, health, nutrition, work

Time, although an intrinsic dimension in our daily lives, is often neglected, especially regarding time devoted to health care. In modern society, there is often insufficient time to fulfill all the demands imposed upon us, impacting our work, social, and family activities, as well as lifestyle. In this scenario, the field of chronobiology is central, providing both a better understanding of the deleterious effects of these changes and approaches for managing these activities. Therefore, chronobiological knowledge is useful in devising preventive measures and interventions.

This special edition sought to bring together scientific evidence addressing chronobiological approaches, involving light exposure, meal times and sleep, as well as their impact on health. We believe the advancement of chronobiological knowledge can help manage time more effectively in today's society, providing new diagnostic and therapeutic techniques in health promotion programs. The studies presented here sought to elucidate, from a chronobiological perspective, the roles of work, sleep, and food intake in the health-disease process. In addition, the ways in which the application of chronobiological knowledge can help in disease treatment and prevention are discussed.

In the experimental model reported by Ren et al., the authors investigated whether time-restricted feeding can facilitate adaptation to a phase advance, given that rhythmic feeding behavior is a non-photic zeitgeber in mammals, especially for peripheral clocks. The authors evaluated both the behavioral adaptation and health status of the mice studied. The experiment involved two groups: one with no phase advance; and the other with a 6-h phase advance, combining different time-restricted feeding approaches. The results suggest that food restriction at the end of the activity phase facilitated circadian adjustment of mice in the phase advance group, whereas the mice in the group without restricted feeding took twice as long to adjust. Concomitantly, the authors found an improvement in resilience to sepsis. These findings contribute to the discussion on chrononutritional approaches as therapy for circadian misalignment and its consequences, since this experimental study confirmed the feasibility of food intake window control as a strategy to minimize circadian misalignment.

Both the potential benefit of napping after lunch and exposure to blue-enriched bright light for improving attention and performance have been widely investigated. However, Zhou et al. compared the effects of a blue-enriched bright light intervention vs. exposure to normal indoor light on the alertness, mood, and performance of undergraduate students who had a habit of napping after lunch, and also investigated whether these effects were task dependent. In a study of 17 healthy undergraduate students (mean age of 20.47 years) who had the habit of napping after lunch (between 13:00 h and 14:00 h), three interventions were applied: (1) a short nap and normal indoor light condition; (2) no nap and normal indoor light condition; and (3) no nap and blue-enriched bright light condition. The results showed that deprivation of habitual post-lunch naps impaired subjective vigilance, mood, and performance on attention and memory activities. Exposure to blue-enriched bright light, however, partially minimized these effects. Thus, the authors suggest that blue-enriched bright light exposure can be a potential chronobiological strategy for people deprived of their usual nap after lunch.

In a quasi-experimental study carried out by Nehme et al., the authors evaluated the impact of the Covid-19 pandemic on the eating habits of day and shift workers, as well as on adherence to a nutritional counseling program, and its effect on food intake and body weight. A sample of 151 workers (77.5% day workers and 22.5% shift workers) was monitored during both pre-pandemic and pandemic periods. The authors found an increase in energy consumption, macronutrients and several micronutrients during the pandemic period relative to the pre-pandemic period. A reduction in body weight was also found for individuals who adhered to the counseling program, while weight gain occurred in those with non-adherence. Adherence to the counseling program also impacted the consumption of proteins and some micronutrients. Interestingly, shift workers tended to consume their afternoon snack earlier during the pandemic compared with the pre-pandemic period, while day workers maintained their usual meal times. Overall, shift workers ate their meals later than day workers. In short, the pandemic had a negative impact by increasing food intake, regardless of work shift, but adherence to the nutritional counseling program was associated with management of food intake and reduced body weight. This study demonstrates the importance of individualizing eating plans, specifically in an atypical difficult situation such as the pandemic period, which has led to shifts in several social and personal behaviors. Furthermore, the importance of the active participation of workers was also highlighted, through focusing on their preferences, routine and chronobiological approaches.

Finally, the last paper in this issue was a retrospective study conducted by Mota et al. evaluating the influence of social jetlag on metabolic parameters and blood pressure in 625 patients with non-communicable chronic diseases (diabetes mellitus 2, systemic arterial hypertension, obesity, and dyslipidemia). A high level of social jetlag was shown to impair the metabolic and blood pressure control of these patients after a 1- year follow-up, especially among patients with impaired glycemic and lipid profiles. Since chronic non-communicable diseases are a global public health problem, understanding how circadian misalignment affects these diseases in the long term is fundamental for their control. The data from the study also underscores the importance of the chronobiological strategy in the treatment of non-communicable chronic diseases, in which regular sleep times are essential to balance social demands and health. In sum, all contributions reveal a broad emerging area that involves a variety of exciting approaches for promoting health.

## Author Contributions

EM wrote the first draft of the present manuscript. CC and CM contributed to its critical revision. All authors contributed to the article and approved the submitted version.

## Conflict of Interest

The authors declare that the research was conducted in the absence of any commercial or financial relationships that could be construed as a potential conflict of interest.

## Publisher's Note

All claims expressed in this article are solely those of the authors and do not necessarily represent those of their affiliated organizations, or those of the publisher, the editors and the reviewers. Any product that may be evaluated in this article, or claim that may be made by its manufacturer, is not guaranteed or endorsed by the publisher.

